# Investigation of Relationship between Hemodynamic and Morphometric Characteristics of Aortas in Pediatric Patients

**DOI:** 10.3390/jcm13175141

**Published:** 2024-08-29

**Authors:** Olga V. Doroshenko, Alex G. Kuchumov, Mikhail V. Golub, Irina O. Rakisheva, Nikita A. Skripka, Sergey P. Pavlov, Yulija A. Strazhec, Petr V. Lazarkov, Nikita D. Saychenko, Roman M. Shekhmametyev

**Affiliations:** 1 Institute for Mathematics, Mechanics and Informatics, Kuban State University, Krasnodar 350040, Russia; oldorosh@mail.ru (O.V.D.); kychymov@inbox.ru (A.G.K.); nktskrpk2000@gmail.com (N.A.S.); nikita-s03@mail.ru (N.D.S.); 2 Biofluids Laboratory, Perm National Research Polytechnic University, Perm 614990, Russia; strazhecjulia@gmail.com; 3 Department of Computational Mathematics, Mechanics and Biomechanics, Perm National Research Polytechnic University, Perm 614990, Russia; riolegovna@yandex.ru; 4 Department of General Anatomy, Kuban State Medical University, Krasnodar 350063, Russia; pavlov_94@mail.ru; 5 S.G. Sukhanov Cardiovascular Center, Perm 614013, Russia; pvlazarkov@gmail.com (P.V.L.); sidjin@gmail.com (R.M.S.)

**Keywords:** aorta, patient-oriented modeling, metamodel, classification, biomechanical features, infant cardiovascular surgery

## Abstract

**Background**: The utilization of hemodynamic parameters, whose estimation is often cumbersome, can fasten diagnostics and decision-making related to congenital heart diseases. The main goal of this study is to investigate the relationship between hemodynamic and morphometric features of the thoracic aorta and to construct corresponding predictive models. **Methods**: Multi-slice spiral computed tomography images of the aortas of patients with coarctation diagnoses and patients without cardiac or vascular diseases were evaluated to obtain numerical models of the aorta and branches of the aortic arch. Hemodynamic characteristics were estimated in key subdomains of the aorta and three branches using computational fluid dynamics methods. The key morphometric features (diameters) were calculated at locations in proximity to the domains, where hemodynamic characteristics are evaluated. **Results**: The functional dependencies for velocities and pressure on the corresponding diameters have been fitted, and a metamodel has been constructed employing the predicted values from these models. **Conclusions**: The metamodel demonstrated high accuracy in classifying aortas into their respective types, thereby confirming the adequacy of the predicted hemodynamic characteristics by morphometric characteristics. The proposed methodology is applicable to other heart diseases without fundamental changes.

## 1. Introduction

A recognized reference method for determining the aortic pressure gradient is catheter-based angiography. Nevertheless, this procedure is invasive and could have several complications [1]. One of the most affordable and safest non-invasive techniques for the coarctation of the aorta (CoA) diagnostics is transthoracic echocardiography (TTE), which uses the Doppler effect to measure the pressure gradient—a clinically significant hemodynamic metric [2]. While a pulsed and continuous wave Doppler provides an investigation of the velocity and direction of blood flow in the thoracic aorta, the color flow Doppler offers the visualization of turbulence in the vicinity of its narrowing [3]. The capacity of TTE to assess associated congenital cardiac and valve abnormalities is a helpful feature [4]. Due to its advantages, TTE is the initial and primary imaging modality for suspected CoA [5]. Nonetheless, the favored contemporary imaging method for the non-invasive diagnosis and monitoring of CoA is multi-slice spiral computed tomography (MSCT) of the heart [6,7,8]. CT angiography with intravascular contrast is the most effective method for the vascular bed visualization in the diagnosis of aortic CoA [9]. It is well known that images should be an integrated component of a cardiovascular abnormalities diagnosis and treatment program [10,11,12,13,14,15]. It is crucial that the interpretation of the images obtained during diagnosis must be performed by a highly qualified expert in the field of congenital heart disease (CHD) [16,17], since the subjectivity of the analysis [18] and the image clarity [19] can negatively affect the patient’s quality of life [20].

This study concerns the CoA, which is CHD characterized by the narrowing of the aorta, resulting from an abnormal junction of the aortic isthmus and the arterial duct. CoA is the fifth most common form of CHD [21] and various estimates of its occurrence range from 5–9% of all CHD [22], appearing in 4.4 cases out of 10,000 live births [23]. It is especially concerning as, if left untreated, CoA might have permanent effects such arterial hypertension [24], left ventricular dysfunction [25] or the creation of aortic and intracranial aneurysms [26]. Patients under 32 years with a CoA diagnosis have a mortality rate as high as 50% and aortic aneurysm rupture accounts for 21% of cases [27,28,29]. Traditional first-line treatment is surgical repair, transcatheter balloon angioplasty and stent implantation [30]. However, extra-anatomical correction is favored if concomitant cardiac surgery is necessary.

Currently, the field of healthcare and biomedical engineering technologies is actively using machine learning (ML) algorithms, which enable the early detection of a variety of patient diseases and the prediction and prevention of their progression [31,32,33,34,35]. The healthcare system can become more digitally advanced, faster and efficient with the use of ML [36,37,38]. However, ML techniques typically necessitate the availability of extensive datasets, which are frequently unattainable in the field of medical research. Bioengineering scientists have to confront the problem of data scarcity, because such medical studies are usually complicated, expensive, and time-consuming, but they also carry hazards to the lives and health of patients [39], clinicians [40] and medical laboratory scientists [41]. It should also be emphasized that medical research must be carried out ethically if it involves the collection and processing of patient personal data [42,43]. The situation for bioengineering scientists is made even more dire by the fact that not all patients are prepared to divulge information about their health status to anyone but care workers. One potential solution to the above mentioned issue is the using of numerical modeling to generate synthetic data, which can be the input data of the ML model [44]. Numerical modeling makes it feasible to reduce the labor, material and time expenses for clinicians. Numerical analysis is widely used to investigate the biomechanical parameters of the human cardiovascular system [45,46,47,48]. A multitude of current therapeutic approaches for different pathological disorders necessitate the development of customized numerical models implemented for specific patients requiring intricate, occasionally operational treatment.

The current literature provides a large number of examples of patient-specific cardiovascular structure models [49,50,51,52,53,54]. The representation of the factual patient’s condition with reliable anatomy is the first step towards building a numerical model and using image-based computational fluid dynamics (CFD) analysis in the field of cardiovascular medicine [55,56,57,58,59,60,61,62,63,64,65,66]. A more comprehensive depiction of the patient’s hemodynamics is offered via a personalized numerical blood flow model created at several scales [67,68]. The utilization of hemodynamic parameters derived from CFD-modeling as input data for ML algorithms [44,69,70,71,72], including classification tasks [73], will facilitate the acceleration in the diagnostics of disease and the planning of intricate clinical procedures. This strategy can help qualified experts to select the most effective pathology treatment procedures, hence lowering the likelihood of medical errors that many patients suffer from [74,75]. It was shown in [76] that insufficient training data have a significant negative impact on classification accuracy. Moreover, it is also established that the accuracy of a classification algorithm is predominantly influenced by the representative nature of a specific dataset, rather than its size [77].

CoA is the pathology that frequently necessitates quick surgical intervention to prevent serious complications. In order to accurately classify the presence or absence of such an aortic disease, it is essential to have a sufficient amount of high-quality baseline data that can be ensured by maximizing the approximation of numerical models to reality. Because of the complicated detailed geometries, patient-individualized boundary conditions, unsteady flow profiles, which cumulatively give physiologically realistic results [78,79], and the need for skilled bioengineering scientist to properly tune the analyzed computational model parameters, the accurate modeling of blood flow dynamics is a labor-intensive task. The accurate and timely diagnosis of CoA can be greatly aided by blood flow quantification. The study of blood flow dynamics now makes significant use of computational fluid dynamics. Computational fluid dynamics is a non-invasive, in silico set of techniques that yield numerical values for the vital blood flow parameters such as velocity and pressure that are essential to hemodynamic research. The employment of CFD simulations makes it easier to calculate critical clinical parameters such as pressure gradient [80], wall shear stress [81], oscillatory shear index [82] and vorticity. It should be mentioned that in order to obtain insight into certain hemodynamic parameters, computational tools are required as they cannot be quantified clinically. In addition to providing a better way to visualize flow dynamics, computational fluid dynamics (CFD) makes it possible to calculate other, hard-to-measure characteristics including wall shear stresses (WSS) and energy loss.

It is known that low WSS is predictive of aortic aneurysm growth and rupture. An analysis of computed results can serve as a quantitative evaluation of such a disease’s progress and development. WSS are also useful in predicting the thrombosis of aortopulmonary shunts and conduits, so this index can also be adopted in a Blalock–Taussig shunt effectiveness evaluation. Moreover, velocity fields are representative of numerous physical attributes, and pressure fields serve as a stand-in for oxygen supply. Rather than using invasive techniques, CFD may evaluate certain extremely useful clinical characteristics, such as cerebral and myocardial oxygen supply and systemic oxygen delivery. Another application of CFD analysis is the simulation and prediction of an intervention’s hemodynamics and results during virtual surgery. The application of CFD analysis has been mostly in preoperative assessment, planning and prediction rather than intra- or postoperative treatment because the process is primarily based on imaging and other hemodynamic data and is frequently time-consuming due to significant data processing times. Simplified numerical 2D [83] and 3D [84] models with CoA imitation were considered, although these models do not focus on the particular patient but rather demonstrate a broad hemodynamic trend.

**Figure 1:**
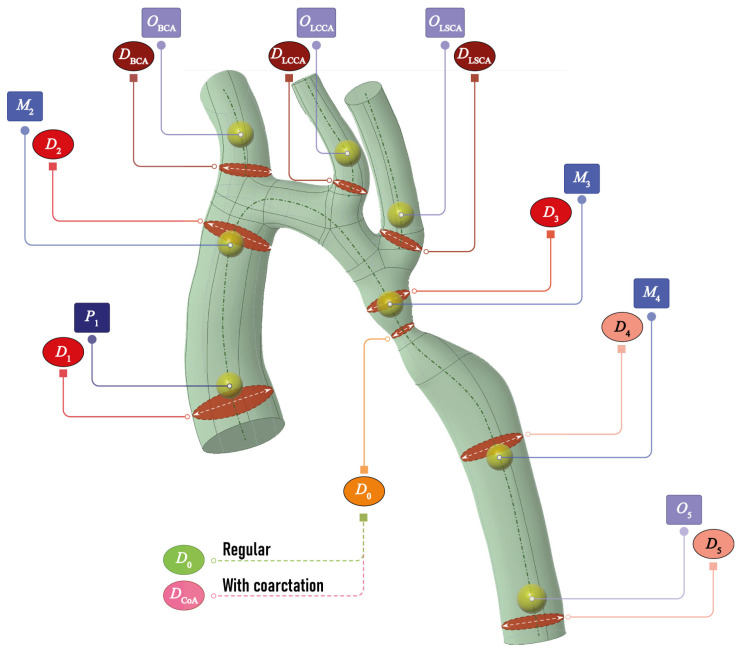
An example of an aorta with markers illustrating positions for measuring morphometric features (diameters) and spheres for evaluating hemodynamic features.

At first, the relationship between hemodynamic and morphometric features of the thoracic aorta is investigated in this study. Such a relationship can be employed to construct a surrogate model or a metamodel, which can peform as a substitute for a computationally expensive CFD model and predict the key hemodynamic characteristics based on morphometric characteristics. For this purpose, eight key sub-domains, where hemodynamic characteristics such as pressure and velocities must be evaluated, have been chosen. The corresponding spherical domains of 1 mm diameter with centers situated at the chosen points are depicted in Figure 1 as yellow spheres, whereas dash-dotted lines show the central lines of the vessels. Hemodynamic characteristics in $P_{1}$ describe the input into the aorta, whereas $O_{i}$ has been situated relatively close to the origins of four vessels—the brachiocephalic artery (BCA), the left common carotid artery (LCCA), the left subclavian artery (LSCA) and the descending part of the aorta—to have data about output flow. Three points $M_{i}$ inside the aorta and distant from the points $P_{1}$ and $O_{i}$ have been added to obtain information on blood flow between the input/output locations.

Nine key locations, where the diameters of the vessels should be measured, have been chosen close to the spheres. The corresponding cross-sections are also exhibited in Figure 1 as crossing ellipses with diameters. The morphometric parameters of the thoracic aorta included the following: the aortic diameter 1 cm above the sinotubular junction ($D_{1}$), the diameter in the area of the transition of the ascending aorta into the arch ($D_{2}$), the diameter in distal arch measurements ($D_{3}$), the diameter of the narrowest part of the descending aorta ($D_{0}$), the diameter of the greatest expansion of the descending aorta ($D_{4}$) and the diameter at the level of the aortic hiatus ($D_{5}$) [85,86,87]. The diameters $D_{\mathrm{BCA}}$, $D_{\mathrm{LCCA}}$ and $D_{\mathrm{LSCA}}$ of the branches of the aortic arch in the area of their origin (DBCA, DLCCA and DLSA) were also measured. In other words, five diameters $D_{1}$, $D_{\mathrm{BCA}}$, $D_{\mathrm{LCCA}}$, $D_{\mathrm{LSCA}}$ and $D_{5}$ describe the morphometry of input and output vessels, whereas four diameters $D_{2}$, $D_{3}$, $D_{0}$, $D_{4}$ reveal the hemodynamic changes inside the aorta. It should be noted that $D_{0}$ is the diameter of the narrowest descending aortic cross-sections starting from the aortic arch, which coincides with the coarctation diameter $D_{0}$ in the case of the corresponding diagnosis.

The MSCT images of thoracic aortas of 30 patients with CoA diagnoses and 30 patients without cardiac or vascular diseases with various clinical data are considered in this study. For each patient, a unique numerical model of the thoracic aorta and its branches is made based on the segmented MSCT images. The utilization of CFD analysis enabled us to determine hemodynamic parameters and then to apply the obtained results to the metamodel construction. This work emphasizes the significance and necessity of developing patient-specific numerical models for hemodynamic analysis and corresponding machine learning methods in order to facilitate the classification of aortic diseases as a surgical planning decision support system.

## 2. Materials and Methods

### 2.1. Patients, MSCT Data Evaluation and Morphometric Features Extraction

#### 2.1.1. Subjects and Data Collection

The current retrospective study was carried out in S.G. Sukhanov Cardiovascular Center, Perm, Russia. Data on 60 infants born from October of 2020 to December of 2023 were collected. Patients with gestational age ranging from 25 to 32 weeks and birth weights ranging from 500 up to 1500 g were included in the study. Newborns with developmental defects (including combined congenital heart defects), with infectious diseases or severe somatic disorders were excluded from the study. CT scans, together with the patient’s history, were used to divide patients into two groups. Written informed consent was obtained from the patients’ parents. The study was approved by the Ethics Committee of S.G. Sukhanov Cardiovascular Center, Perm, Russia.

#### 2.1.2. MSCT Data Evaluation

In order to obtain the hemodynamic characteristics via CFD simulations, the data are saved into .stp format, which is required for loading in ANSYS, used for CFD simulations. Some details on the used CDF model are given in Section 2.2.1. To convert the MSCT images for each patient into 3D models suitable for CFD simulations using ANSYS, the segmentation and corresponding evaluation were performed, resulting in the approximated vessel surfaces; for more details, see [55]. Such a process provides us with the array of points laying at the approximated surfaces of the vessels in .stl format, which is used for the numerical automatic extraction of morphometric key features employing the algorithm proposed in [55]. At the same time, morphometric features of the aorta and three arteries have been measured manually using RadiAnt DICOM Viewer software (Version 2022.1.1, Medixant, Poland). The data obtained from the manual measurements were used for cross-checking with the results of the automatic evaluation of the morphometric characteristics.

**Figure 2:**
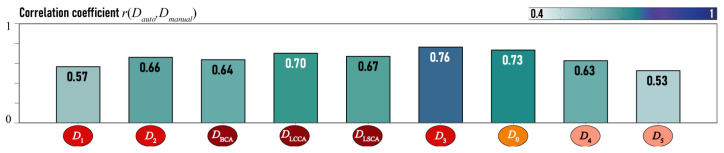
Correlation coefficient $r(D_{\mathrm{auto}},D\mathrm{manual})$ for manually and automatically evaluated diameters.

The correlations between the estimates of the morphometric characteristics calculated using both methods, manual and automatic, are shown in Figure 2. A certain discrepancy between the estimations is observed due to a slightly different choice of the points on the aorta vessel, where the manual and automatic method were made using each method. Furthermore, even experienced personnel cannot precisely determine the minimal cross-section, which is calculated using the numerical algorithm [55]. The best agreement of the data is observed at the points that define the diameter in the region of the transition from the arch to the descending part of the aorta $D_{3}$ and the diameter of the greatest narrowing of the descending part of the aorta $D_{0}$. The determination of points $D_{1}$ and $D_{5}$ is the most different between manual and automatic evaluation. However, it should be noted that the minor discrepancies in diameter measurements do not significantly affect the analysis of the aorta.

#### 2.1.3. Morphometric Features

**Figure 3:**
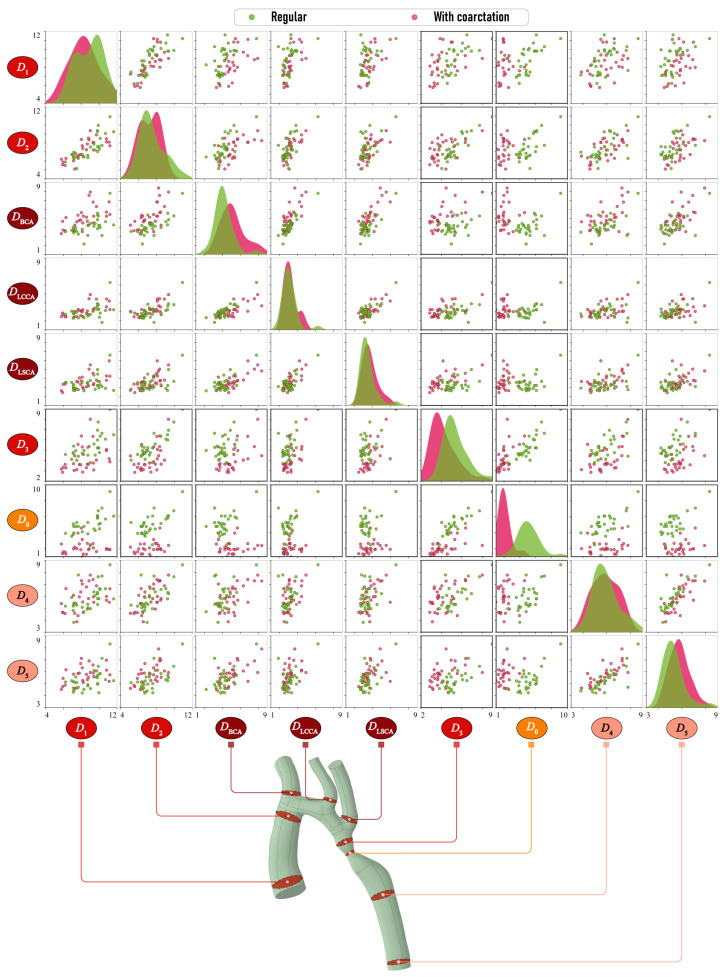
Scatter plots demonstrating pairwise relationships between cross-section diameters $D_{i},i=\bar{1,5}$, $D_{\mathrm{BCA}}$, $D_{\mathrm{LCCA}}$, $D_{\mathrm{LSCA}}$ and $D_{\mathrm{CoA}}$, in mm.

Figure 3 demonstrates pairwise relationships between the cross-section diameters $D_{i},i=\bar{0,5}$, $D_{\mathrm{BCA}}$, $D_{\mathrm{LCCA}}$ and $D_{\mathrm{LSCA}}$ obtained after processing the surfaces of vessels evaluated at the previous stage, as described in Section 2.1.2. Here, green and pink colors are used to distinguish between the results for normal aortas and aortas with CoA, respectively. Each scatter plot displays a set of points in Cartesian coordinates, where the value of the first variable conditions the position on the horizontal axis, while the position on the vertical axis is determined by the value of the second variable. The separation of the points on the scatter plots into clusters suggests that the characteristics on which they are plotted are of significant importance in determining the condition of the aorta.

A clearly visible separation can be seen for the parameter $D_{0}$, which is quite obvious. There is also a separation for the parameters $D_{BCA}$ and $D_{3}$, although it is not as clear. On the diagonal are plotted Kernel Density Estimates (KDE), a non-parametric method used to estimate the probability density function and to visualize the continuous representation instead of the discrete histogram. The separation of the KDE plots also indicates the significant separate capability of the corresponding morphometric characteristic. The KDE plots for $D_{0}$ demonstrate that the values for the diameters of the aortas with CoA are, on average, smaller than those of normal aortas. Additionally, the spread of values for normal aortas is observed to be larger. The diameters $D_{3}$ of aortas exhibiting CoA are also smaller, yet the variability of the values is approximately equivalent to that observed in normal aortas. That is, there is an equal probability of the occurrence of the values in question in both the considered aortic types. In the case of diameter $D_{BCA}$, a greater degree of variability is observed in aortas with CoA, as well as larger values, on average, compared with normal aortas.

### 2.2. CFD Model and Hemodynamic Features

#### 2.2.1. CFD Model Application

Blood flow is considered an incompressible Newtonian fluid flow with a constant density of 1060 kg/m^3^ and a dynamic viscosity of 0.005 Pa·s. We used an incompressibility equation and Navier–Stokes equations to describe fluid flow:

(1) ∇·v=0,

(2) $$\rho \frac{\partial v}{\partial t}+\rho \left(v\cdot \nabla \right)v=\nabla \cdot \sigma ,$$

(3) σ=pI+τ.

Here v is the fluid velocity, σ is the stress tensor, ρ is the fluid density, *p* is the pressure, I is the identity tensor and τ is the extra stress tensor.

At the aorta inlet, the time dependence of velocity for the cardiac cycle was used. For the aorta outlets, the time dependence of pressure on the time computed using the 0-D model was used. The mathematical model describing blood circulation includes 13 differential equations and several dozen algebraic relations. By transformations, it is possible to express all variables through unknowns, which allows us to pass to a system of 13 differential equations with 13 unknowns. The constants and the solution of the system of equations are described in the paper [62]; for more details related to the model construction, see [65].

The calculations were performed in the ANSYS CFX hydrodynamic module of ANSYS. The inlet, all the outlets and the walls of the aorta are specified in the design modeler after the particular geometry of the aorta (surfaces of vessels) is loaded into ANSYS. The mesh is then overlaid using the Body Sizing tool, specifying the type and size of the mesh element, and the Inflation tool, which allows us to increase the density of the mesh in the wall regions to control wall effects. Blood flow is then modeled from the aortic inlet to each of the 4 outlets. The control pressure is set to 1 atm, whereas the fluid flow is simulated for 1 s with time steps of 0.005 s; 2 heart beats are simulated during this time.

**Figure 4:**
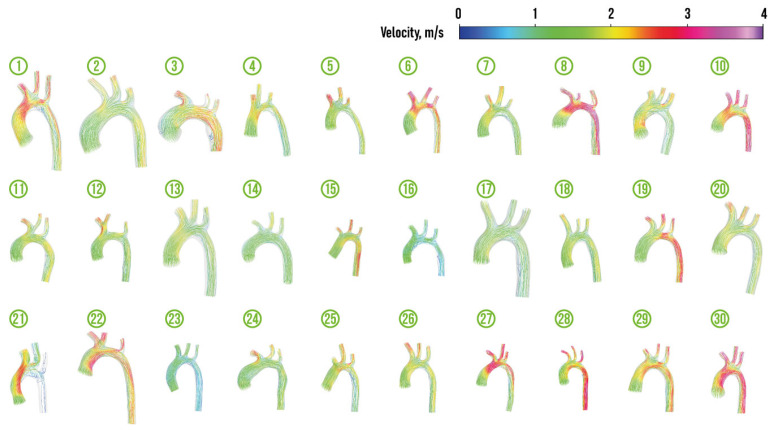
Velocity streamlines in regular aortas from the prepared dataset.

**Figure 5:**
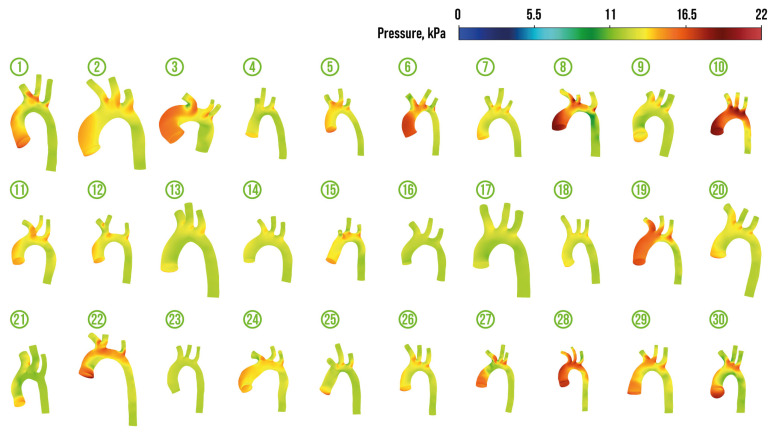
Pressure distribution in regular aortas from the prepared dataset.

**Figure 6:**
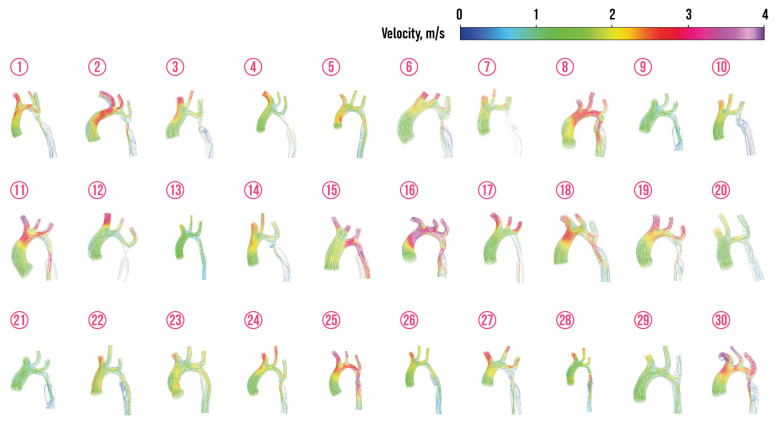
Velocity streamlines in aortas with CoA diagnosis from the prepared dataset.

**Figure 7:**
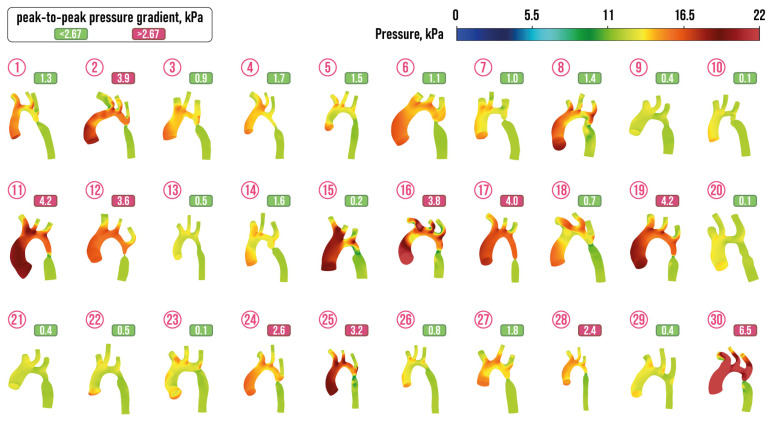
Pressure distribution in aortas with CoA diagnosis from the prepared dataset.

Figure 4, Figure 5, Figure 6 and Figure 7 depict velocity streamlines and pressure distribution in regular aortas and aortas with CoA diagnosis from the prepared dataset. The colors of the velocity streamlines and pressure correspond to the value, which can be evaluated using colorbars situated at the top right of these figures. All the depicted aortas with arteries are drawn using the same scale.

The visualization of the dataset demonstrates the natural fact that blood flow into the descending aorta is not hampered in regular aortas (the only exception is regular aorta 21). It should be noted that the presence of a common brachiocephalic trunk (the origin of both common carotid arteries and the right subclavian artery from a single trunk that originates from the aortic arch) was detected in two cases (regular aortas 11 and 12). This type of origin can occur in 10% of cases and is considered as the normal type, because it is not associated with cardiovascular diseases [88,89].

#### 2.2.2. Hemodynamic Features Extraction and Dataset Formation

Velocities and pressure distributions were computed using CFD tools. Velocity and flowrate as a quantified amount of blood volume moving in a time unit is a general parameter useful in the evaluation of all anomalies and repairs. Pressure, which can be considered as a surrogate for the energy generated by the heart in the circulation, is also a valuable parameter important in all anomalies and repairs. Additionally, we computed wall shear stress, which is important for the prediction of thrombosis of aortopulmonary shunts and conduits. The employment of the data on pressure and velocities for all the points of the model in ANSYS is impractical, since the total number of points exceeds 10,000. Therefore, pressure and velocities were calculated at 850–900 points inside the chosen spherical domains ($P_{1}$, $O_{i}$ and $M_{i}$) with centers situated at the chosen points, as depicted in Figure 1. The averaged values of velocities and pressure in domain Ω are denoted as v(Ω) and p(Ω), respectively. At the end, the dataset containing information on morphometric features (diameters and central lines) and hemodynamic characteristics (velocities and pressure) is prepared.

It was observed that the values of velocity and pressure at the selected points of the aorta were proved to have a high degree of homogeneity. Consequently, the mean values of velocity and pressure at each point were identified as discrete hemodynamic characteristics. The peak-to-peak pressure gradient, which is the difference in peak pressure proximal and beyond the narrowed segment, is often used as an indicator of severity of the CoA, when there is significant collateral circulation [21,90]. Thus, ACC/AHA guidelines for adults with congenital heart disease recommended the following interpretation of the peak-to-peak CoA gradient γ: patient with γ>20 mm Hg = 2.67 kPa requires surgical intervention [91]. The peak-to-peak CoA gradient

$$\gamma =p\left(M_{3}\right)-p\left(M_{4}\right)$$

is depicted for 30 considered aortas with the CoA in Figure 7.

**Figure 8:**
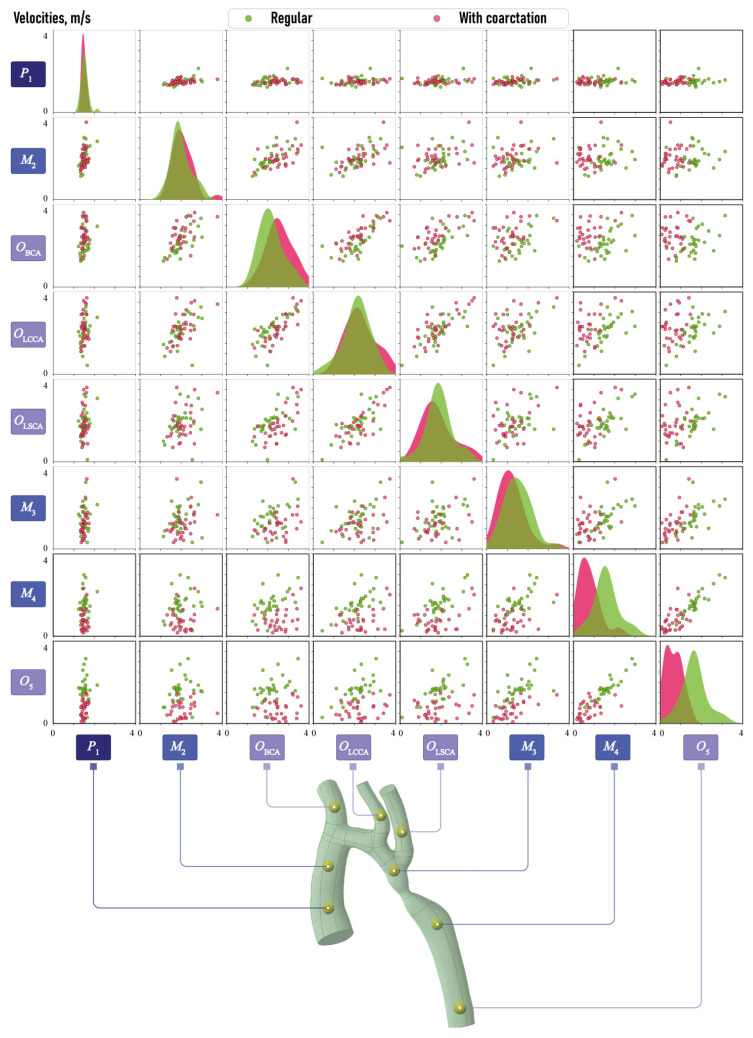
Scatter plots demonstrating pairwise relationships between averaged velocities calculated in critical domains $P_{1}$, $O_{\mathrm{BCA}}$, $O_{\mathrm{LSCA}}$, $O_{\mathrm{LSCA}}$, $M_{2}$, $M_{3}$, $M_{4}$ and $O_{5}$.

**Figure 9:**
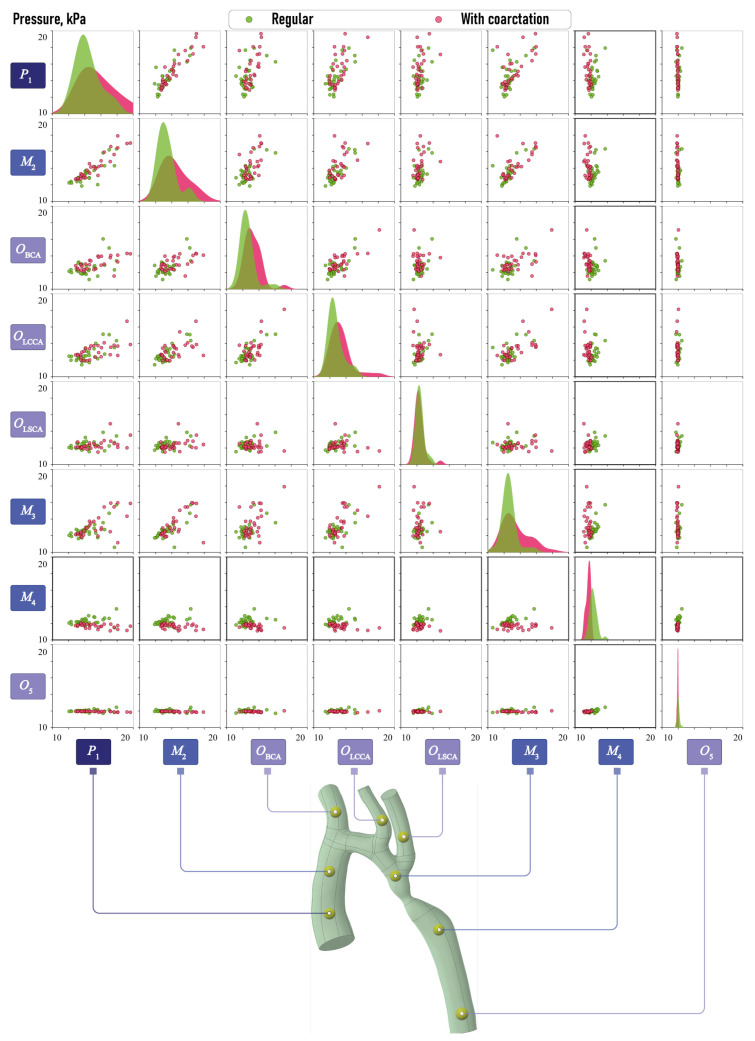
Scatter plots demonstrating pairwise relationships between averaged pressure calculated in critical domains $P_{1}$, $O_{\mathrm{BCA}}$, $O_{\mathrm{LSCA}}$, $O_{\mathrm{LSCA}}$, $M_{2}$, $M_{3}$, $M_{4}$ and $O_{5}$.

Figure 8 and Figure 9 illustrate the pairwise relationships in the aortic velocity dataset and in the aortic pressure dataset, as well as the Kernel Density Estimation (KDE) functions. Similarly of morphometric characteristics, the green color denotes normal aortas, while the pink color represents aortas with CoA. The greatest data separations by clusters in the plots by aortic type are observed for velocities at points $M_{3}$ and $O_{5}$, with a less pronounced separation at point $O_{\mathrm{BCA}}$. A clear separation into clusters is not evident in terms of aortic point pressure values, in contrast to the clear separation observed in terms of velocity. It can only be noted that at points $P_{1}$, $M_{1}$$O_{\mathrm{BCA}}$ and $O_{\mathrm{LSCA}}$, there is a larger dispersion of pressure in aortas with coarctation and a shift of KDE towards higher values. At points $O_{\mathrm{LSCA}}$ and $O_{5}$, the pressure is observed to be practically identical for both types of aortas. At point $O_{5}$, the dispersion of the values is sufficiently minimal that the pressure value can be considered to be constant. At the $M_{3}$ point, the KDE plot in aortas with coarctation shifts towards smaller values.

### 2.3. Extraction of Key Hemodynamic and Morphometric Characteristics

#### 2.3.1. Morphometric Features

**Figure 10:**
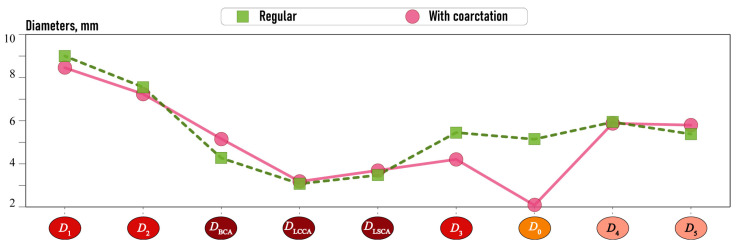
Mean of the estimated diameters.

The mean values of the diameters under consideration for normal aortas and aortas with CoA are presented in Figure 10. The graph demonstrates that diameters $D_{0}$, $D_{3}$ and $D_{1}$ in aortas with CoA are smaller than in normal aortas, while diameters $D_{BCA}$ and $D_{5}$ are larger. The remaining diameters have minimal variation between the two aorta types.

In order to ascertain the discriminative quality of the selected morphometric features, a two-group classification has been used. The binary logistic regression model

(4) $$\begin{array}{c} z=\alpha_{0}+\alpha_{1}D_{1}+\alpha_{\mathrm{BCA}}D_{\mathrm{BCA}}+\alpha_{3}D_{3}+\alpha_{4}D_{0}+\alpha_{5}D_{5},\\ P\left(z\right)=\frac{1}{1+e^{-z}} \end{array}$$

is defined via the probability P(·) of the occurrence of the event under consideration. Here, *z* is a linear combination of the diameters with unknown coefficients $\alpha_{i}$. If the estimated probability is above the threshold, the aorta is classified as an aorta with CoA; otherwise, it is classified as a normal one. The threshold is typically set at 0.5.

According to the practice of model training, the available data are randomly divided into training and test sets in an 80:20 ratio [92]. The logistic regression coefficients of Equation (4) calculated using the training set are presented in Table 1. In accordance with expectations, the diameter $D_{0}$ plays the most significant role in the aortic classification process. Nevertheless, the diameter $D_{3}$, despite its notable variation between aortas with and without CoA, does not impact the efficacy of the classification procedure. The latter can be attributed to the high correlation between diameters $D_{0}$ and $D_{3}$, which makes the inclusion of both parameters redundant. Though diameter $D_{1}$ makes a minor contribution to the classification model, its exclusion from the model degrades classification quality. Furthermore, it is noteworthy that this model exhibits 100% classification accuracy on the test dataset.

#### 2.3.2. Hemodynamic Features

**Figure 11:**
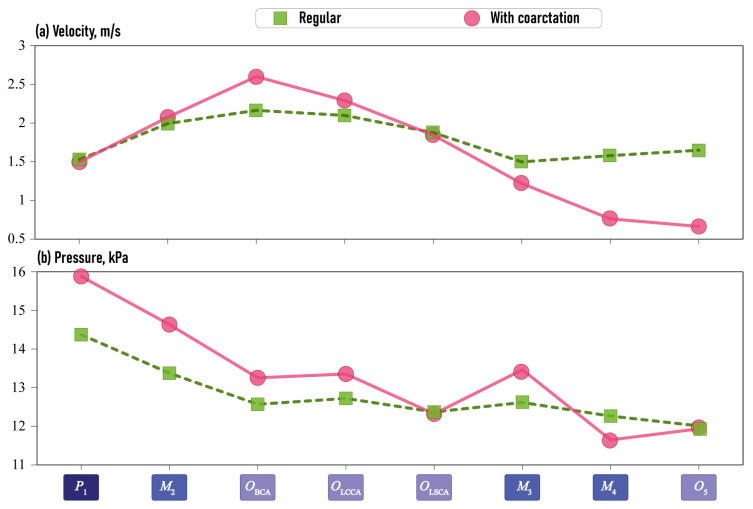
Mean values of the calculated hemodynamic characteristics at the points.

In order to analyze the discrepancy in hemodynamic characteristics between normal aortas and aortas with CoA, the mean values of velocity and pressure are plotted in Figure 11. The analysis confirms the inferences visible in Figure 8 and Figure 9. Above all, it can be observed that there is a velocity reduction in $M_{3}$ and $O_{5}$ after constriction in the aortas with CoA. Furthermore, an increase in velocities is observed in $O_{\mathrm{BCA}}$, situated in the brachiocephalic artery. This elevation in velocities may be due to the narrowing of the descending aorta. Velocities in the left common carotid artery and left subclavian artery are comparable between the two aortic types. The primary discrepancy in pressure variation occurs in $P_{1}$ of the input into the aorta and in the intermediate domain $M_{1}$ of the ascending aortic arch. In the case of aortas with CoA, the pressure is typically higher. Additionally, elevated pressure is observed in the brachiocephalic artery in $O_{\mathrm{BCA}}$, in the left common carotid artery in $O_{\mathrm{LCCA}}$ and the domain $M_{2}$ situated before aorta narrowing, while a decrease is observed in $M_{3}$ located after aorta narrowing. However, this discrepancy is relatively minor, and the pressure at these points is not a key feature for aortas with CoA.

The classification is performed based on the selected key hemodynamic characteristics and employs logistic regression to analyze and assess their capacity to differentiate between groups. The logistic regression model for hemodynamic features is defined as follows:

(5) $$y=P\left(\alpha_{0}+\alpha_{1}v\left(O_{\mathrm{BCA}}\right)+\alpha_{2}v\left(M_{3}\right)+\alpha_{3}v\left(O_{5}\right)+\alpha_{4}p\left(P_{1}\right)+\alpha_{5}p\left(M_{1}\right)\right).$$

Here, P(·) is again the probability calculated using Equation (4). The estimated probability $\hat{y}$ of belonging to the class of aortas with CoA is compared to a threshold defined at the 0.5 level.

The coefficients in logistic regression given by Equation (5) calculated using the training set as described above are presented in Table 2. The averaged velocities observed in domain $M_{4}$ after the narrowing of the aorta provide the most significant contribution to the function used in the classification procedure. It should be noted that though the averaged pressure in $P_{1}$ and $M_{2}$ only slightly affects classification outcomes, its absence leads to a notable decline in the classifier quality.

The constructed classification routine demonstrated an accuracy rate of 0.83 in the test set. Namely, one normal aorta was incorrectly identified as an aorta with CoA. A similar error was observed with one aorta with CoA, representing one error in six. It should be noted that the classifier using the complete set of hemodynamic features provides a lower accuracy (0.75).

## 3. Results

### 3.1. Metamodel and Relationships between Hemodynamic and Morphometric Characteristics

#### 3.1.1. Hemodynamic Features Prediction by Morphometric Characteristics

It is known that the volume flow rate of blood entering at a certain section of a vessel between two of the cross-sections is related to a change in the diameter of the vessel. The relation between the velocities and diameters of the input and output vessels can be formulated in the form of a balance equation written in terms of squares of diameters and velocities [93]. Therefore, quadratic dependencies have been considered in addition to linear models.

The most crucial hemodynamic features under consideration are the velocities in the descending aorta (domain $O_{5}$ and $M_{4}$) and the velocity in the brachiocephalic artery (domain $O_{\mathrm{BCA}}$). In view of the correlation coefficients of 0.77 and 0.74 between the velocity at $O_{5}$ and $O_{\mathrm{BCA}}$ and the pressure in $P_{1}$, respectively, the initial step is to construct a linear regression model describing the relationship between $p(P_{1})$ and the morphometric parameters. The following regression estimation has been made:

(6) $$\begin{array}{c} \hat{p}\left(P_{1}\right)=11.14+0.34D_{1}-0.29D_{\mathrm{BCA}}-0.55D_{0}+0.79D_{5},\\ R^{2}=0.224,\;\;p-\mathrm{level}=0.00839. \end{array}$$

Despite the relatively low coefficient of determination, the regression model (6) is statistically significant. The pressure $p(P_{1})$ increases together with aorta diameters $D_{1}$ and $D_{5}$, and decreases in conjunction with the increase in the diameter of the brachiocephalic artery in the absence of a coarctation. Since $p(M_{2})$ is also a key hemodynamic characteristic, the corresponding regression model was also estimated:

(7) $$\begin{array}{c} \hat{p}\left(M_{2}\right)=11.2+0.45D_{2}-0.27D_{BCA}-0.53D_{0}+0.47D_{5},\\ R^{2}=0.255,\;p-\mathrm{level}=0.00319 \end{array}$$

Regression in the form of Equation (7) is also statistically significant and behaves similar to the model described by Equation (6). The only difference between these models is the employment of the $D_{2}$ diameter instead of the $D_{1}$ diameter in Equation (6), which corresponds to the part of the aorta where the pressure $p(M_{2})$ is calculated.

The functional dependencies for velocities on diameters of the aorta and outgoing arteries were fitted according to the balance equation proposed in [93]. Given the significant correlation between velocities and pressure $p(P_{1})$, the prediction $\hat{p}\left(P_{1}\right)$ calculated employing the model described by Equation(6) has been added to the equations. Accordingly, the estimate of regression for the $v(O_{\mathrm{BCA}})$ depending only on the significant parameters has the following form:

(8) $$\begin{array}{c} \hat{v}\left(O_{\mathrm{BCA}}\right)=-0.42+0.07{\left(\frac{D_{1}}{D_{\mathrm{BCA}}}\right)}^{2}-0.27{\left(\frac{D_{0}}{D_{\mathrm{BCA}}}\right)}^{2}+0.1{\left(\frac{D_{4}}{D_{\mathrm{BCA}}}\right)}^{2}+0.17\hat{p}\left(P_{1}\right),\\ R^{2}=0.23,\;\;p-\mathrm{level}=0.00695 \end{array}$$

Equation (8) connecting the velocity $v(O_{\mathrm{BCA}})$ and the diameter in the brachiocephalic artery is described using three key parameters: the diameter of the ascending part of aorta, the diameter of the narrowest part of the descending aorta and the diameter corresponding to the dilation of the descending aorta, normalized to the diameter of the brachiocephalic artery. If the diameter $D_{0}$ increases, indicating the absence of coarctation, the velocity in the brachiocephalic artery decreases. The balance equation does not include a pressure variable. However, the introduction of $\hat{p}\left(P_{1}\right)$ into the regression model results in a sufficient improvement in the coefficient of determination $R^{2}$.

The regression models for the velocities $v(M_{4})$ and $v(O_{5})$ have been constructed in the same manner model (8). In this process, only those diameters that most accurately describe the velocities were retained. A summary of the estimated parameters of these regression models is provided below.

(9) $$\begin{array}{c} \hat{v}\left(M_{4}\right)=1.67+0.39{\left(\frac{D_{1}}{D_{4}}\right)}^{2}-0.87{\left(\frac{D_{\mathrm{BCA}}}{D_{4}}\right)}^{2}-2.17{\left(\frac{D_{LCCA}}{D_{4}}\right)}^{2}+0.58{\left(\frac{D_{3}}{D_{4}}\right)}^{2}-0.04\hat{p}\left(P_{1}\right),\\ R^{2}=0.421,\;\;p-\mathrm{level}=0.00002 \end{array}$$

(10) $$\begin{array}{c} \hat{v}\left(O_{5}\right)=1.7+0.29{\left(\frac{D_{1}}{D_{5}}\right)}^{2}-0.87{\left(\frac{D_{\mathrm{BCA}}}{D_{5}}\right)}^{2}-1.25{\left(\frac{D_{\mathrm{LCCA}}}{D_{5}}\right)}^{2}+0.75{\left(\frac{D_{3}}{D_{5}}\right)}^{2}-0.06\hat{P}\left(P_{1}\right),\\ R^{2}=0.521,\;p-\mathrm{level}<0.00001 \end{array}$$

The velocities are measured in the thoracic aorta after constriction. As a result, models described by Equations (9) and (10) present a similar appearance. It should be noted that the velocities in the aorta are independent of the diameter of the aortic constriction $D_{0}$, as expected. Instead, they are positively dependent on the diameter $D_{3}$, which is measured before being compared to $D_{0}$. An increase in the diameter of $D_{BCA}$ in the brachiocephalic artery and the diameter of $D_{LCCA}$ in the left common carotid artery allows for the redirection of blood flow in the case of a coarctation, resulting in velocity reduction. All diameters included in the regression models are normalized to the diameter of the part of the aorta where the velocity is measured. The pressure $p(P_{1})$ provides a minor but significant contribution to the accuracy of velocity predictions in the models described by Equations (9) and (10).

**Figure 12:**
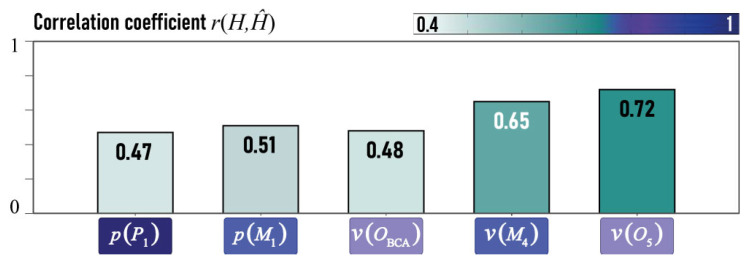
Correlation coefficient $r(H,\hat{H})$ for manually and automatically evaluated diameters.

All the constructed models provide a linearization of complex physical dependencies and, therefore, have low coefficients of determination, yet they are statistically significant. In order to ascertain the degree of correspondence between the predicted hemodynamic characteristics $\hat{H}$ and the data obtained from measurements in a complex numerical model *H*, the calculated correlation coefficients $r(H,\hat{H})$ are depicted in Figure 12. It should be noted that $v(O_{5})$ is the most accurately predicted velocity by the models. The velocity $v(O_{BCA})$ is averaged in the predictions, and the model cannot predict rare variations in velocity. It can be posited that the models are effective at predicting hemodynamic characteristics.

### 3.2. Metamodel

At the end, a metamodel is created, employing the predictive models constructed in Section 3.1.1. Metamodels should confirm the quality of the predicted values of key hemodynamic characteristics. While the predicted values may not be as comprehensive as the ones obtained from CFD modeling, they should be able to separate aortas by class. A linear logistic regression-based classifier was constructed based on the predictions obtained from the models described by Equations (6)–(10).

(11) $$y=P\left(\alpha_{0}+\alpha_{1}\hat{v}\left(O_{\mathrm{BCA}}\right)+\alpha_{2}\hat{v}\left(M_{3}\right)+\alpha_{3}\hat{v}\left(O_{5}\right)+\alpha_{4}\hat{p}\left(P_{1}\right)+\alpha_{5}\hat{p}\left(M_{1}\right)\right).$$

The results of the estimation of logistic regression coefficients for Equation (11) are presented in Table 3. It can be concluded that all estimates of hemodynamic characteristics contribute significantly to the classification of aortas.

The quality of classifier presented by Equations (11) is evaluated on the same test dataset used in classifier (5), with CFD modeling data replaced by the data from models described by Equations (6)–(10). Only 1 aorta out of 12 in the test set under consideration was incorrectly classified: one aorta with CoA was erroneously categorized as a normal one. The classifier (11) demonstrates a higher accuracy (0.92) than classifier (5), which provides 0.83.

## 4. Discussion and Concluding Remarks

In this paper, a relationship between hemodynamic and morphometric characteristics has been investigated using simple machine learning methods. Morphometric characteristics are widely used by clinicians, since they are easily measurable on MSCT images employing various toolkits, allowing for a quite accurate determination of anomalies in aortic development. In turn, hemodynamic characteristics provide a better understanding of the patient’s condition. However, the determination of these characteristics is more cumbersome. For instance, the implementation of CFD simulations used in this study is computationally expensive and requires engaging an expert in CFD.

The sufficient simplification of this procedure can be achieved using a surrogate model or a metamodel, which can substitute for a more complicated CFD model. Such a metamodel is described in Section 3.2 for two considered classes of aortas (with coarctation diagnoses and without cardiac or vascular diseases). The constructed models has allowed to predict velocities and pressure in some arteries and the descending aorta rather accurately. Though these classes can be distinguished relatively easily, the presented approach can be extended for other kinds of deceases of aortas. Moreover, the extension of the dataset should allow us to apply neural networks to perform the predictions with even higher accuracy.

The demonstrated promising outcomes open prospects for the extension of the proposed approach to more medically complex clinical cases. The proposed approach will be further used for the evaluation of the Blalock–Taussig shunt position in patients with congenital heart disease [94]. The idea is to evaluate morphometric features of patients and to use the ML technique to predict how Blalock–Taussig shunt will affect new-born hemodynamics without direct CFD or fluid structure interaction simulation. The approach presented in this manuscript can be considered as the first important step to achieve this goal. The performed intravascular contrast measurements do not provide enough data for the precise investigation of pulmonary atresia and the tetralogy of Fallot based on the obtained MSCT images. The proposed methodology can be applied to these diseases; however, the latter demands some changes in the choice of the proper location of the points for measuring hemodynamic and morphometric characteristics. Moreover, additional vessels should be included in the CFD model in the case of pulmonary atresia.

## Figures and Tables

**Table 1:** Estimated logistic regression coefficients in Equation (4).

${\hat{\alpha }}_{1}$	${\hat{\alpha }}_{\mathrm{BCA}}$	${\hat{\alpha }}_{3}$	${\hat{\alpha }}_{0}$	${\hat{\alpha }}_{5}$
−0.399	0.999	0.11	−1.726	1.134

**Table 2:** Logistic regression coefficients of Equation (5).

Characteristic	$v(O_{\mathrm{BCA}})$	$v(M_{3})$	$v(O_{5})$	$p(P_{1})$	$p(M_{1})$
Coefficients	1.377	−1.472	−1.914	0.482	0.414

**Table 3:** Logistic regression coefficients of Equation (11).

Characteristic	$\hat{v}\left(O_{\mathrm{BCA}}\right)$	$\hat{v}\left(M_{3}\right)$	$\hat{v}\left(O_{5}\right)$	$\hat{p}\left(P_{1}\right)$	$\hat{p}\left(M_{1}\right)$
Coefficients	−1.05	−1.05	−1.05	1.138	0.799

## Data Availability

The Russian Ethics Review Authority only granted publication of aggregated data, which means that individual data cannot be shared.

## Abbreviations

The following abbreviations are used in this manuscript:

CoA	coarctation of the aorta
TTE	transthoracic echocardiography
CHD	congenital heart disease
MSCT	multi-slice spiral computed tomography
ML	machine learning
CFD	computational fluid dynamics
BCA	brachiocephalic artery
LCCA	left common carotid artery
LSCA	left subclavian artery
